# Clinical and prognostic significance of follicular helper and regulatory T cells in bladder cancer draining lymph nodes

**DOI:** 10.1038/s41598-024-70675-1

**Published:** 2024-09-02

**Authors:** Zahra Mansourabadi, Ali Ariafar, Nooshafarin Chenari, Hossein Hakimellahi, Yasmin Vahidi, Zahra Faghih

**Affiliations:** 1 Shiraz Institute for Cancer Research, School of Medicine, Shiraz University of Medical Sciences, P.O. Box: 7134845550, Shiraz, Iran; 2 Department of Immunology, School of Medicine, Iran University of Medical Sciences, Tehran, Iran; 3 Department of Urology, School of Medicine, Shiraz University of Medical Sciences, Shiraz, Iran; 4 Department of Molecular and Translational Medicine, University of Brescia, Brescia, Italy

**Keywords:** Bladder cancer, Draining lymph node, Tfh, TFR, Tumour immunology, Urological cancer

## Abstract

Follicular helper and regulatory T cells (Tfh/TFR) cells are distinct subsets of CD4^+^ cells that have been recognized for their critical role in regulating cellular reactions within the germinal centers of lymphoid follicles. In the present study, we aimed to determine the presence and the frequency of these cells in draining lymph nodes of patients with bladder cancer (BC). Forty-six patients with BC who had undergone radical cystectomy and pelvic lymph node dissection were enrolled. Following routine pathological examination, a portion of the dissected lymph nodes was minced to obtain a single-cell suspension. Mononuclear cells were then separated using Ficoll-Hypaque gradient centrifugation, and the samples with proper viability (> 95%) were subjected to further analysis. To phenotype the follicular subsets, cells were stained with appropriate fluorochrome-conjugated antibodies specific for CD4, CXCR5, BCL6, and FOXP3. The cells were then acquired on a four-color flow cytometer. The data were analyzed with the FlowJo software version 10.8.1 package. Our analysis indicated that, on average 37.89 ± 16.36% of CD4^+^ lymphocytes in draining lymph nodes of patients with BC expressed CXCR5. The majority of them were negative for FOXP3, representing helper subsets (28.73 ± 13.66). A small percent simultaneously expressed BCL6 transcription factor (1.65% ± 1.35), designated as Tfh (CD4^+^BCL6^+^CXCR5^+^FOXP3^-^). While less than 10% of CD4^+^ lymphocytes expressed CXCR5 and FOXP3, 1.78 ± 2.54 were also positive for BCL6, known as TFR. Statistical analysis revealed that the frequency of both Tfh and TFR cells was higher in draining lymph nodes of patients with tumor-infiltrated nodes (P = 0.035 and P = 0.079, respectively) compared to those with negative ones. The percentage of these cells was also higher in high-grade tumors compared to low-grade ones (P = 0.031 for both). Our data collectively indicated that however approximately one third of CD4^+^ lymphocytes expressed CXCR5 and accordingly had the capacity to enter the follicles, less than 2% of them represented Tfh and TFR phenotypes. The percentage of these cells increased in progressed tumors and showed an association with negative prognostic factors.

## Introduction

T follicular helper (Tfh) cells as a distinct subset of CD4^+^ T cells, are primarily recognized within the germinal centers (GCs) of lymphoid follicles; playing a critical role in regulating cellular reactions and development of humoral immunity. These cells are identified by the expression of cell surface molecules such as CXC-chemokine receptor 5 (CXCR5), co-inhibitory receptor programmed cell death 1 (PD-1), B cell lymphoma 6 (BCL-6), IL-21, and inducible T cell costimulator (ICOS), largely facilitate their interactions with B cells^1,2^. Their pivotal role in adaptive immunity places them at the intersection of various immunological processes. Over the past decade, significant attention has been given to Tfh cells from the initial evidence of their presence in human lymphoid tissues. The fascination with Tfh cells largely originates from the discovering BCL6 as an indispensable transcription factor for Tfh cells and GC formation, the main site for B cell affinity maturation^3,4^. Accordingly, the regulation of Tfh cells is essential for GC responses to generate and select B cells with heightened affinities^5^.

Immune responses within secondary lymphoid organs, particularly Tfh cells, and germinal center reactions are modulated by a specialized subset of regulatory cells, named T follicular regulatory (TFR) cells. They predominantly reside within the germinal centers, and crucially contribute to the maintenance of immune homeostasis, prevention of autoimmunity, and regulation of humoral immunity. Any alterations in their abundance or function can lead to a range of auto-antibody-associated diseases^6,7^. Such insights hold promising implications for the development of new therapies targeting autoimmune diseases and cancer^8,9^.

Emerging research has also suggested a role for Tfh/TFR cells in the development and progress of several types of cancers, including lymphoma, breast cancer, colorectal cancer, non-small cell lung cancer (NSCLC), and hepatocellular carcinoma^10–15^. However, the involvement of Tfh and TFR cells in bladder cancer (BC) has been poorly understood. Accordingly, in the present study, we aimed to elucidate the presence and the frequency of these subsets in draining lymph nodes of patients with BC.

## Materials and methods

### Patients

Forty-six patients with BC underwent radical cystectomy and pelvic lymph node dissection were enrolled in the study. Following routine pathological examination, a part of the dissected lymph nodes immersed in the culture medium was sent to the laboratory. Infiltrated tumor cells in draining lymph nodes were histologically determined by expert pathologists as node positive. The clinical and pathological information of patients was extracted from medical records. Ethical approval was obtained from the Shiraz University of Medical Sciences ethics committee (IR.SUMS.REC.1395.221).

### Isolation of mononuclear cells from lymph nodes

Single-cell suspensions were prepared by mincing fresh lymph nodes into small pieces in complete culture media, RPMI 1640 (Biosera, France) containing 10% fetal bovine serum (Gibco, USA) and 1% penicillin/streptomycin (Biosera). The cells were then filtered through a 40-μm cell strainer (SPL, Korea), and mononuclear cells were separated using Ficoll-Hypaque (Biosera) gradient centrifugation. The mononuclear ring was harvested, washed twice, and dissolved in 1 × phosphate-buffered saline (PBS). The viability of the cells was checked with trypan blue exclusion assay (Biosera), and the samples with proper viability (> 95%) were subjected to further analysis.

### Cell staining and flow cytometry analysis

To phenotype follicular subsets, the mononuclear cells were first surface-stained with appropriate fluorochrome-conjugated antibodies (all from BioLegend, USA) specific for CD4 (PercP, clone: SK3) and CXCR5 (Alexa Fluor® 647, clone: RF8B2) (Table 1). The concentration of antibodies was based on the manufacturer's recommendations. For intracellular staining, the cells were fixed and permeabilized with FOXP3 Buffer set according to the manufacturer's instruction (BD Biosciences, USA). The cells were then stained for the transcription factors FOXP3 (PE, clone: 259D; BioLegend) and BCL6 (Alexa Fluor® 488, clone: K112.91; BD Biosciences). The cells were washed, re-suspended in 1 × PBS, and acquired on a four-color flow cytometer (FACSCalibur, BD Biosciences). Single-stained controls containing cells stained individually with each fluorochrome-conjugated antibody (FITC, PE, PerCP, and APC) were used to control the fluorescence signal spillover and to create the compensation matrix. Concentration of antibodies was based on the manufacturer's recommendations. After applying the compensation matrix, a minimum of 100 × 10^3^ events was recorded, and the data were analyzed using FlowJo software version 10.8.1 (BD Life Sciences).

**Table 1:** Antibody characteristics used for flow cytometry analysis.

Human antigen	Fluorochrome	Clone	Company
BCL-6	Alexa Fluor® 488	K112.91	BD Biosciences
FOXP3	PE	259D	BioLegend
CD4	PercP	SK3	BioLegend
CXCR5	Alexa Fluor® 647	RF8B2	BioLegend

### Phenotype determination of follicular subsets

**Fig. 1:**
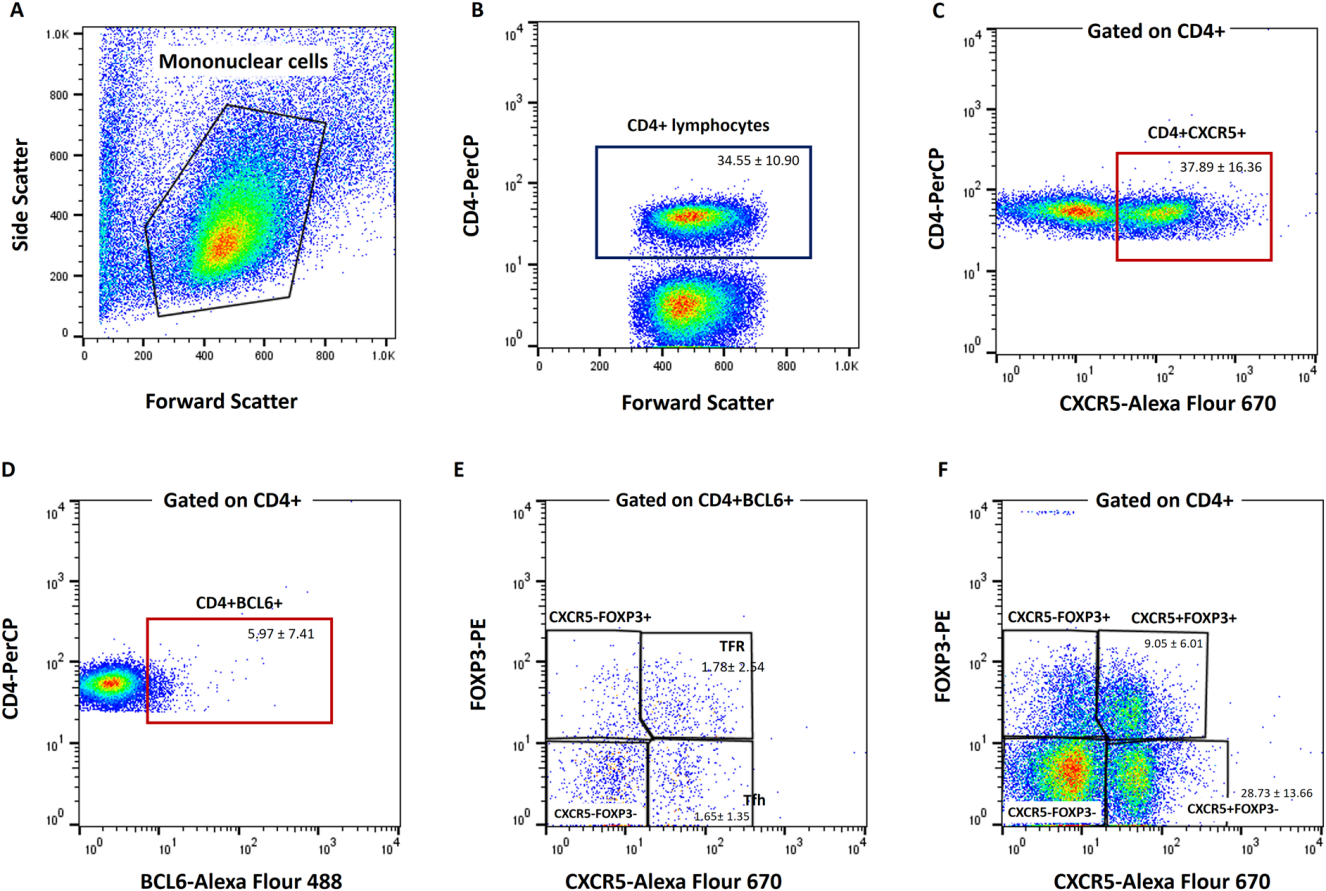
Phenotype determination of different CD4^+^ follicular subsets in tumor draining lymph nodes from patients with bladder cancer. To determine the frequency of various follicular helper and regulatory subsets, CD4^+^ cells (**B**) were first selected within the mononuclear/lymphocyte gate (**A**). Besides determining the total frequency of CD4^+^CXCR5^+^ (**C**), and CD4^+^BCL6^+^ (**D**) lymphocytes, a subset of CD4^+^BCL6^+^ cells with low or no expression of FOXP3 were considered as Tfh cells; those cells positive for FOXP3 were identified as TFR cells (**E**). FOXP3^–^ or FOXP3^+^CXCR5^+^CD4^+^ irrespective of BCL6 expression was also assessed (**F**). *Tfh* T follicular helper, *TFR* T follicular regulatory.

To determine the frequency of distinct subsets of follicular helper and regulatory cells, as illustrated in Fig. 1, CD4^+^ cells were first isolated (1B) within the lymphocyte gate (1A). The phenotypes of different subsets were then defined based on the expression patterns of BCL6, CXCR5, and FOXP3. In addition to determining the total frequency of CD4^+^CXCR5^+^ (1C), and CD4^+^BCL6^+^ cells (1D), CD4^+^ lymphocytes that were BCL6^+^CXCR5^+^ with minimal or no FOXP3 expression were categorized as Tfh cells (1E). The cells with positive expression of FOXP3 were identified as TFR cells, denoted as FOXP3^+^BCL6^+^CXCR5^+^CD4^+^ lymphocytes (1E). We also considered related subsets with analogous phenotypes (FOXP3^-^CXCR5^+^CD4^+^ and FOXP3^+^CXCR5^+^CD4^+^) irrespective of BCL6 expression (1F). The frequency of each subset was then determined in the CD4^+^ population which is reported in Table 2.

**Table 2:** Frequency of different follicular subsets of CD4^+^ in tumor draining lymph nodes of patients with bladder cancer.

Cell subset	Min	Max	Median	Mean ± SD
CD4^+^ lymphocytes	15.10	56.60	35.80	34.55 ± 10.90
CD4^+^ CXCR5^+^	1.86	92.90	39.05	37.88 ± 16.36
CD4^+^ BCL6^+^	0.54	45.90	3.85	5.97 ± 7.41
Helper subsets				
Tfh: CD4^+^BCL6^+^CXCR5^+^FOXP3^–^	0.07	6.77	1.42	1.65 ± 1.35
CD4^+^CXCR5^+^FOXP3^–^	0.88	82.50	28.05	28.73 ± 13.66
CD4^+^CXCR5^–^FOXP3^–^	5.62	88.40	52.65	51.28 ± 16.08
CD4^+^BCL6^+^CXCR5^-^FOXP3^-^	0.04	2.73	0.55	0.75 ± 0.66
Regulatory subsets				
CD4^+^Foxp3^+^	1.53	53.80	16.30	19.55 ± 10.70
TFR: CD4^+^BCL6^+^FOXP3^+^CXCR5^+^	0.19	14.10	1.06	1.78 ± 2.54
CD4^+^CXCR5^+^FOXP3^+^	0.27	25.90	9.40	9.05 ± 6.01
CD4^+^CXCR5^-^Foxp3^+^	0.12	51.90	6.56	9.86 ± 9.01
CD4^+^BCL6^+^CXCR5^–^FOXP3^+^	0.02	42.10	0.29	1.59 ± 6.17

*Tfh* T follicular helper, *TFR* T follicular regulatory.

### t‐distributed stochastic neighbor embedding (t‐SNE) analysis

To find unbiased phenotypically distinct subpopulations, we used the FlowJo software with its plugins, t‐distributed stochastic neighbor embedding (t‐SNE), and Phenograph, as the unsupervised machine learning algorithms. To perform the analysis, flow cytometry standard (FCS) downsampled files were concatenated into a single FCS file to generate a comprehensive dataset for analysis. The t-SNE plugin was then run with the default values (perplexity = 20, learning rate = 200, iterations = 1000, and theta = 0.5); followed by the Phenograph plugin (v.2.4) with K = 30. The FSC, SCC, CD4, CXCR5, BCL6, and FOXP3 parameters were entered into the analysis.

### Statistical analysis

All data were analyzed with SPSS version 23 (SPSS GmbH Software, Germany). Nonparametric Mann–Whitney *U* and Kruskal–Wallis H tests were used to compare the frequency of subsets between two or more groups, respectively. P values less than 0.05 (two-tailed) were considered statistically significant. The graphs were depicted by GraphPad Prism software (GraphPad Software, Boston, Massachusetts USA).

### Ethics declarations

All methods were performed in accordance with the guidelines and regulations of the Declaration of Helsinki. The study was approved by the Ethics Committee of Shiraz University of Medical Sciences (IR.SUMS.REC.1395.221). Participants were fully informed about the study, and informed consent was obtained from all patients.

## Results

### Clinical and pathological characteristics of the patients

Following pathology confirmation, 46 lymph nodes from patients with BC were included in the final analysis. The mean age of patients was 63.93 ± 12.09 years. According to the pathological reports, urothelial carcinoma was the most frequent tumor type accounting for 97.8% (45/46) of cases. Thirteen patients had at least one involved lymph node (designated as LN^+^ patients; 28.3%). Most patients were in stage III (24/46, 53.3%). The main clinicopathological characteristics of the patients are summarized in Table 3.

**Table 3:** Clinicopathological characteristics of patients with bladder cancer.

Characteristics		N (valid percentage)
Age		63.93 ± 12.09
Gender	Male	37 (80.4)
	Female	9 (19.6)
Tumor type	Urothelial carcinoma (UC)	45 (97.8)
	Non-UC	1 (2.2)
Histological grade	Low	3 (6.5)
	High	43 (93.5)
T-stage	T1	4 (8.9)
	T2	24 (53.3)
	T3	7 (15.6)
	T4	10 (22.2)
	Unreported	1
Lymph node involvement	Positive	13 (28.3)
	Negative	33 (71.7)
N-stage	N0	33 (71.7)
	N1	3 (6.5)
	N2	9 (19.6)
	N3	1 (2.2)
TNM-stage	I	3 (6.7)
	II	18 (40)
	III	24 (53.3)
Organ-confined tumor (OCT)	OCT	25 (54.3)
	non-OCT	21 (45.7)
Muscle invasion	Positive	41 (91.1)
	Negative	4 (8.9)
	Unreported	1
Perivesical fat invasion	Positive	15 (33.3)
	Negative	30 (66.7)
	Unreported	1
Lymphovascular invasion	Positive	17 (38.6)
	Negative	27 (61.4)
	Unreported	2
Perineural invasion	Positive	25 (58.1)
	Negative	18 (41.9)
	Unreported	3
Tumor necrosis	Positive	20 (64.5)
	Negative	11 (35.5)
	Unreported	15
Tumor shape	Papillary	27 (84.4)
	Flat (sessile)	5 (15.6)
	Unreported	14

### Association of follicular helper and regulatory subsets with bladder cancer clinical and pathological parameters

Our analysis indicated that on average 37.89% ± 16.36% of CD4^+^ lymphocytes in draining lymph nodes of patients with BC expressed CXCR5, indicating their capacity to enter the follicles. The majority of these cells (28.73% ± 13.66%) were negative for FOXP3, representing helper subsets. A small percentage of CD4 + cells simultaneously expressed CXCR5 chemokine receptor and BCL6 transcription factor (1.65% ± 1.35), designated as Tfh in our study (CD4^+^BCL6^+^CXCR5^+^FOXP3^-^). While less than 10% of CD4^+^CXCR5^+^ lymphocytes expressed FOXP3, with 1.78% ± 2.5% were also positive for BCL6, known as TFR.

**Fig. 2:**
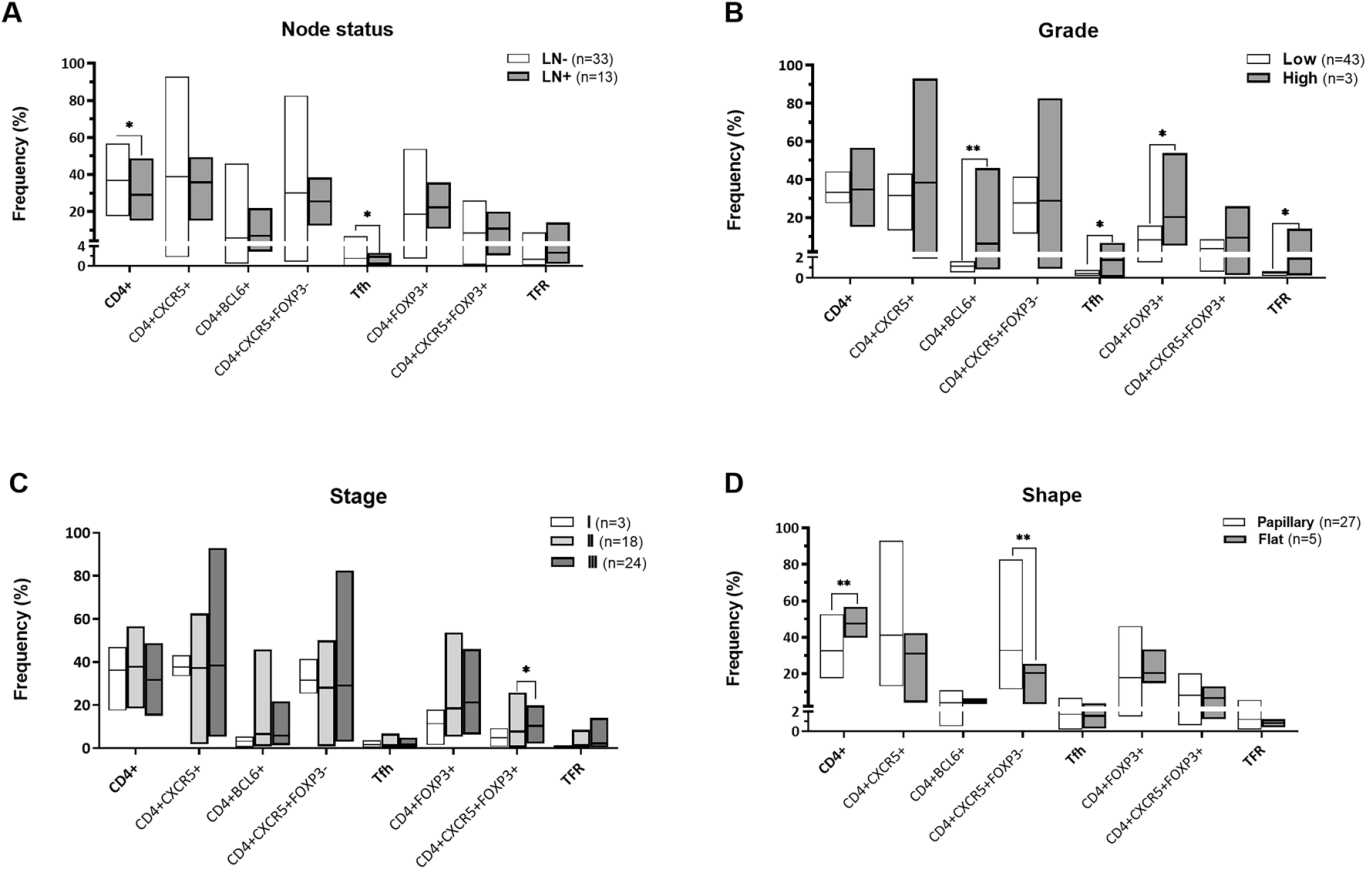
Association of CD4^+^ follicular subsets with clinicopathological characteristics of bladder cancer (n = 46). Follicular helper and regulatory subsets in bladder cancer draining lymph nodes showed significant variations among patients with different statuses of regional draining node involvement (**A**), histological grade (**B**), stage (**C**), and tumor shapes (**D**). Nonparametric Mann–Whitney *U* and Kruskal–Wallis *H* tests were used to compare the frequency of subsets between two or more groups, respectively. The data are presented as median. *Significant at the 0.05 level (2-tailed), **Significant at the 0.01 level (2-tailed). *Tfh* T follicular helper, *TFR* T follicular regulatory.

We then compared the percentage of various helper and regulatory follicular subsets among patients with different clinical and pathological characteristics (Fig. 2). Surveying for CD4^+^ cells irrespective of subtypes revealed that the frequency of these cells was significantly higher in draining lymph nodes of patients with free nodes (P = 0.019) (Fig. 2A), organ-confined (P = 0.027), and lymphovascular invasion negative tumors (P = 0.031), compared to those with tumor-infiltrated nodes, non-organ confined and invasion positive tumors, respectively.

Regarding follicular subsets, patients with positive nodes showed significantly higher frequency of Tfh cells compared to those with negative ones (P = 0.035) (Fig. 2A). Further analysis revealed their increase in N2 patients compared to those with free nodes (P = 0.030). The percentage of these cells (P = 0.031) and CD4^+^BCL6^+^ cells (P = 0.003) was also higher in high-grade tumors compared to low-grade ones (Fig. 2B). Similar results were observed for regulatory subsets, FOXP3-expressing CD4^+^ cells (P = 0.047) and their follicular subset, TFR (P = 0.031) (Fig. 2B) which showed a significant increase in patients with high-grade tumors. These cells along with CD4^+^CXCR5^+^FOXP3^+^ cells were also more frequent in non-organ confined tumors compared to organ-confined ones (P = 0.056, P = 0.083, and P = 0.013, respectively). TFR cells also tended to increase in patients with at least one tumor-infiltrated node (LN^+^; P = 0.079), patients with stage III (P = 0.077), and tumors with perivesical fat invasion (P = 0.044) compared to those with free nodes, stage II, and negative invasion, respectively. However, their related subset with CD4^+^CXCR5^+^FOXP3^+^ phenotype was higher in stage III compared to patients with stage I and II (P = 0.057 and P = 0.033, respectively) (Fig. 2C). These cells were also higher in patients whose cancer spread outside the bladder (T4) compared to patients with tumors spreading to connective tissue (T1) and the muscle layer (T2) (P = 0.024 and P = 0.066, respectively). Additionally, a significant increase in the frequency of non-follicular CD4^+^CXCR5^-^FOXP3^+^ cells was observed in node positive patients compared to node negative ones (P = 0.010). Further analysis revealed their increase in N2 patients compared to free nodes (P = 0.028). A subset with CD4^+^BCL6^+^CXCR5^-^FOXP3^+^ phenotype was also higher in high-grade patients compared to those with low-grade (P = 0.036) (Fig. 2B).

We further calculated the Tfh/TFR ratio and observed that this ratio was significantly higher in patients with T1 (P = 0.025), T2 (P = 0.025), and T3 (P = 0.027) in comparison to those with T4. Additionally, Tfh/TFR ratio was significantly higher in patients with organ-confined tumors compared to those with non-organ confined tumors (P = 0.016).

The frequency of CD4^+^ lymphocytes and their follicular subsets also showed significant variations in draining lymph nodes of patients with different tumor shapes. Specifically, CD4^+^ lymphocytes (P = 0.003) and CD4^+^BCL6^+^CXCR5^-^FOXP3^-^ cells (P = 0.001) were higher in flat-shaped tumors, whereas CD4^+^CXCR5^+^FOXP3^-^ subsets showed an increase in papillary tumors (P = 0.009). No further significant associations were observed with other clinical and pathological conditions including sex, age, and muscular invasion.

### Heterogeneous subsets with distinct expression of CD4, CXCR5, FOXP3 and BCL6 detected by unsupervised analysis

**Fig. 3:**
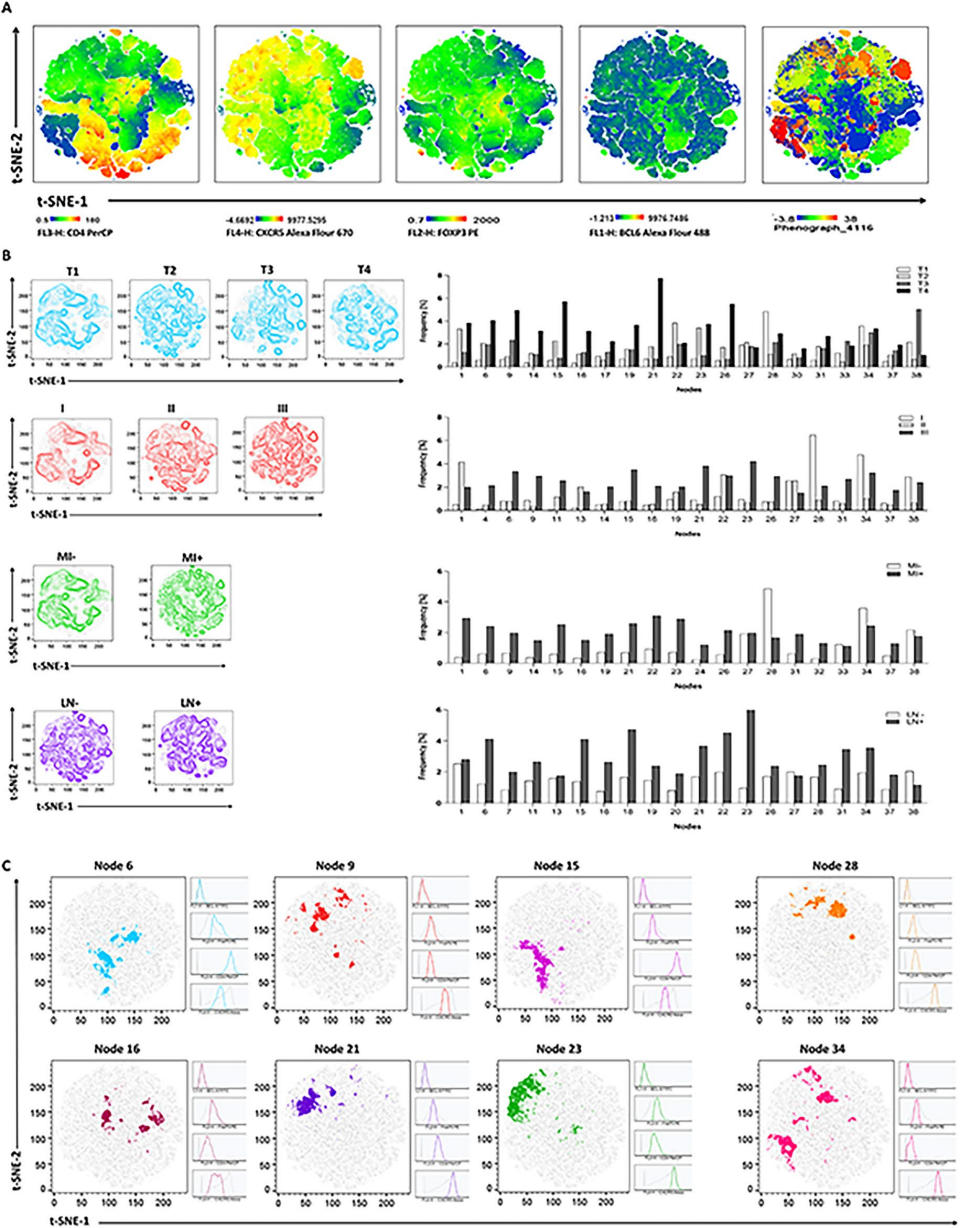
Unbiased gating approach with the t-SNE and Phenograph algorithms. The figure provide a comprehensive visualization and analysis of immune cell subsets in draining lymph nodes of patients with bladder cancer, illustrating their heterogeneity, distribution across clinical parameters, and associations with disease progression and prognosis. (**A**) depicts representative t-SNE map visualizations of CD4, CXCR5, FOXP3, and BCL6 markers in draining lymph nodes of patients with bladder cancer, from left to right. These visualizations illustrate the spatial distribution and expression patterns of these markers across heterogeneous subsets identified through unbiased analysis. The last diagram (left) shows on the distribution of 38 unique clusters identified using Phenograph analysis t-SNE map. Each cluster represents distinct combinations of CD4, CXCR5, FOXP3, and BCL6 expression profiles. (**B**) demonstrates the distribution of spatially resolved populations among patients categorized by T-group, stage, invasion of tumors into the muscle layer, and regional draining nodes status. The bar charts depict the distribution of the 20 most frequent clusters identified by Phenograph within each category. (**C**) Specific clusters (6, 9, 15, 16, 21, and 23) are shown to increase in frequency with tumor progression. These clusters exhibit similar expression patterns of CXCR5, FOXP3, and BCL6, but vary in CD4 expression from negative to high levels. Conversely, clusters 28 and 34 are associated with good prognosis, characterized by high expression of CXCR5 alongside low to negative expression of FOXP3 and BCL6.

To gain deeper insights into follicular T cells within draining lymph nodes in BC, we employed t‐SNE as an additional analytical tool to overcome the limitations of sequential two-dimensional dot plots analysis. Phenograph analysis identified 38 unique clusters based on the defined parameters (FSC, SCC, CD4, CXCR5, BCL6, and FOXP3), in the draining lymph nodes of patients with BC. The clusters were then categorized based on the main clinicopathological parameters. The distribution of the most frequent clusters (20 clusters) in each category was then compared among patients with different T-grouping, stage, muscle invasion, and lymph node involvement. As illustrated in Fig. 3, clusters 6, 9, 15, 16, 21 and 23 showed an increased frequency with tumor progression, including higher T-group, stage III, muscle invasion, and lymph node involvement. These populations exhibited similar expression of CXCR5, FOXP3, and BCL6, but varied in CD4 expression (ranging from negative to high expression). In contrast, clusters 28 and 34 showed a positive association with good prognosis, including lower T-group (T1), stage I, no muscle invasion, and free nodes, with a consistent expression pattern across all markers. These two clusters exhibited high expression of CXCR5 alongside low to negative expression of FOXP3 and BCL6 (Fig. 3C).

## Discussion

In the present study, we investigated two specialized subsets of follicular T cells, Tfh and TFR, in draining lymph nodes of patients with BC. However, our analysis indicated that while approximately one third of CD4^+^ lymphocytes expressed CXCR5 and thus had the capacity to enter the follicles, less than 2% of CD4^+^ lymphocytes expressed BCL6, representing Tfh and TFR phenotypes. The percentage of these cells showed an association with negative prognostic factors and increased in progressed tumors.

Tfh cells are recognized as a crucial subset of effector T cells in orchestrating immune responses in lymphoid tissues. Through the expression of CXCR5, these T cells are able to localize within B cell follicles, thereby providing vital support for B cells^16^. Our data revealed that more than 35% of CD4^+^ lymphocytes in draining lymph nodes of patients with BC expressed CXCR5, potentially enabling their entry into follicles probably through interaction with its ligand, CXCL13. They were predominantly negative for FOXP3, the specific transcription factor of regulatory T cells, and a small fraction (less than 2%) simultaneously expressed BCL6, the linage specific transcription factor of Tfh cells. The frequency of these cells showed an association with BC poor prognosis factors including infiltration of tumor cells into regional nodes, high-stage and -grade tumors. Dysregulation of Tfh cell function or the over- or under-expression of their associated molecules has been observed in various immunodeficiencies or autoimmune diseases^9,17^. In cancer settings, while a role has been proposed for these cells as the origin of some hematologic malignancies, studies on solid tumors are limited. However, in the majority of studies, contrary to our observations, Tfh cells have been correlated with enhanced immune response against cancer, improved clinical outcomes, and increased responsiveness to therapy^15^. Using animal models and human cancers, it has been shown that an active Tfh response in the tumor microenvironment could provide an immune pressure against tumors by activating and linking the main arms of the adaptive immune system, B cells, and cytotoxic T cells, boosting class-switched plasma cells and tumor-specific antibody production^15^. In the BC setting, similarly, Goubet et al. introduced the circulating and tumor-resident Tfh cells as one of the prominent targets for neoadjuvant therapy with Pembrolizumab (anti-PD-1) in muscle-invasive patients. Pembrolizumab seemed to preferentially bind to effector Tfh and activate them to release CXCL13. Most of these cells had a memory phenotype and along with their cognate B cells, were specific for common commensals i.e. *E. coli*. Following treatment, the frequency of these Tfh cells tended to decrease, probably due to their homing to tumor microenvironment where Tfh cells preferentially resided in the tertiary lymphoid structures, linking with cytotoxic CD8^+^ T cells and orchestrating a prolonged immune response against relapse^18^. On the other hand, concordant to our results, Jiang et al. through investigating the hub genes, identified Tfh cells as independent prognostic factors in BC, however, they did not further explore the potential mechanisms^19^. It has been also demonstrated that the proportion of circulating Tfh among CD4^+^ T cells was associated with Binet stages in chronic lymphocytic leukemia (CLL), implying that these cells may be linked to disease prognosis. In addition, when Tfh cells were co-cultured with nurse-like cells, CD40L expression on Tfh cells was down-regulated and accelerated the disease progression^20^. No or negative association of Tfh cells was also observed in the peripheral blood and tumor-draining lymph nodes of patients with breast and lung carcinoma^21,22^.

Tfh cells are supposed to be regulated by special regulatory cells named follicular regulatory T (TFR) cells. Thereby, these cells play an essential role in regulating the GC responses, especially by regulating the proliferation and functions of Tfh and B cells. They have follicular markers in common with Tfh cells including PD-1, CXCR5, CXCL13, BCL6, as well as regulatory cells i.e. FOXP3. We observed that around 20% of CD4^+^ lymphocytes expressed FOXP3 and exhibited regulatory phenotypes, nearly half of these cells seemed to reside in the follicles as they also expressed CXCR5. However, just 2% of CD4^+^ cells simultaneously expressed BCL6; representing TFR phenotype. Similar to Tfh cells, higher frequencies of FOXP3 expressing CD4^+^ cells and their follicular subsets, including TFR, were observed in advanced tumors with higher grades and stages, non-organ confined tumors, and those tumors with positive infiltration to draining nodes. Based on our knowledge, this is the first study reporting TFR in draining nodes of BC. However, emerging data have recently highlighted a role for dysregulation of TFR cells in the pathogenesis of autoimmune diseases and alloreactivity, their functions and phenotypes in cancer are elusive. Given the observation that with tumor progression, the frequencies of TFR cells and other regulatory subsets increase along with Tfh cells, this could imply an immunosuppressive effect exerted by tumors on the function of Tfh cells, thereby affecting B cells and anti-tumor humoral immune responses.

## Conclusion

To the best of our knowledge, this is the first report on follicular T cells in draining lymph nodes of patients with BC. Our data collectively indicated that although approximately one third of CD4^+^ lymphocytes expressed CXCR5 and had the capacity to enter the follicles, less than 2% of them represented Tfh and TFR phenotypes. The percentage of these cells increased in progressed tumors and showed an association with negative prognostic factors in BC. The decrease in the ratio of Tfh/TFR, along with increased frequencies of TFR cells and other regulatory subsets with tumor progression, suggests an immunosuppressive effect exerted by tumors on Tfh cells and thereby on B cells and further anti-tumor humoral immune responses. However, more research is required to determine the exact function of Tfh and TFR cells in BC responses and providing prospective immunotherapeutic targets for BC immunotherapy.

## Data Availability

All data generated or analyzed during this study are included in this published article.
